# Supplementary material for: Evaluating Associations Between Drought and West Nile Virus Epidemics: A Systematic Review
Source: Microorganisms. 2025 Dec 15;13(12):2851. doi: 10.3390/microorganisms13122851 (PMC12736350; doi:10.3390/microorganisms13122851)
Supplement: Supplementary file 1 [file microorganisms-13-02851-s001.zip › Supplemental_info.Drought_WNV_revised_clean.pdf]

Supplemental Material

Table S1. Database Search Terms

**Database**	**Strategy**	**Results**
Web of Science Core Collection	TS=((“West Nile" OR WNV OR "Kunjin virus" OR "Egypt 101 virus" OR “neuro-invasive” OR neuroinvasive OR encephalitis OR meningitis OR "brain inflammation") AND (drought* OR “soil moisture” OR “water scarcity” OR ((arid OR dry OR drier) AND (**weather** OR period* OR condition* OR environment)) OR ((less OR reduced OR low OR lower OR deficit OR deficits) AND (precipitation* OR rainfall OR moisture OR humidity))))<br><br>**Filters:** English	582
PubMed with tags	(("West Nile"[tw] OR West Nile virus[Mesh] OR WNV[tw] OR "Kunjin virus"[tw] OR "Egypt 101 virus"[tw] OR "neuro-invas*" [tw] OR neuroinvas*[tw] OR encephalitis[tw] OR encephalitis[Mesh] OR meningitis[tw] OR meningitis[Mesh] OR "brain inflammation"[tw] AND (drought*[tw] OR droughts[Mesh] OR "soil moisture"[tw] OR "water scarcity"[tw] OR ((arid[tw] OR dry[tw] OR drier[tw]) AND (**weather[tw]** OR period*[tw] OR condition*[tw] OR environment[tw])) OR ((less[tw] OR reduced[tw] OR low[tw] OR lower[tw] OR deficit*[tw]) AND (precipitation*[tw] OR rainfall[tw] OR moisture[tw] OR humidity[tw] OR humidity[Mesh])))))<br><br>**Filters:** English	374

Table S2. Guidelines for Evaluating Study Quality

Selection	Good/Definitely Low Risk of Bias<br>• Study area is clearly defined and all at-risk members of population in that study area are included<br>• Minimal concern for selection bias based on description of subject identification<br>• Exclusion and inclusion criteria specified and would not induce bias
	Adequate<br>• All at-risk members of population in that study area are likely to be included<br>• Enough of a description of the subject identification process to be comfortable that there is no serious risk of bias<br>• Inclusion and exclusion criteria specified and would not induce bias
	Deficient<br>• Little information on subject identification process and/or sampling framework<br>OR

	• Aspects of the subject identification process, selection strategy, sampling framework, or participation raise the potential for bias (e.g., healthy worker effect, survivor bias)
	Critically Deficient<br>• Population selected in such a way that selection bias is likely (For example, only a subset of all at-risk individuals is included, and characteristics of those included differ from those excluded.)
Exposure	Good/Definitely Low Risk of Bias<br>• Includes a direct measure of drought, such as Normalized Difference Water Index (NDWI), Water Table Depth (WTD), soil moisture, wetland connectivity, land surface wetness, ponding frequency, evapotranspiration index, relative humidity, or Palmer Drought Severity Index (PDSI)
	Adequate<br>• Reports a specific time period that indicates the duration of exposure to drought
	Deficient<br>• Exposure is due to season, and season is correlated with drought (e.g. wet vs. dry season)
	Critically Deficient<br>• Exposure is seasonal, but season categories are not direct indicators of drought
Outcome	Good/Definitely Low Risk of Bias<br>• Cases of West Nile neuroinvasive disease or West Nile fever have been diagnosed by a medical doctor or healthcare professional.<br>OR<br>• Cases are based on hospital admissions for patients exhibiting symptoms of neuroinvasive disease
	Adequate<br>• Case data are based on results of a survey that was conducted systematically, without any major sources of bias
	Deficient<br>• Case data are based on results of a survey that was poorly conducted (e.g. only a subset of the exposed population was included in the survey, or the survey was conducted more than 2 months after the main exposure event)
	Critically Deficient<br>• Method not reported
Confounding	Good/Definitely Low Risk of Bias<br>• Clear delineation of how and why confounders were chosen<br>• Covariates may include: temperature, precipitation, patient age, day of the week, holidays<br>• Descriptive information on average number of human cases per week or month is presented<br>• Consideration of unmeasured/residual confounding and its potential effects in the discussion section
	Adequate<br>• Clear delineation of how and why confounders were chosen<br>OR

	• Considers multiple adjustment models in a clearly delineated fashion<br><br>• Covariates may include: temperature, season, age<br>• Descriptive information on average number of human cases per week or month is presented<br>• Consideration of unmeasured/residual confounding and its potential effects in the discussion section
	Deficient<br>• Strategy of evaluating confounding is unclear or is not recommended (e.g., based on statistical significance criteria only)<br>OR<br>• Descriptive information on average number of human cases per week or month is not presented<br>OR<br>• There is likely unmeasured or uncontrolled confounding based on the included confounders and their measurement
	Critically Deficient<br>• No adjustment for confounding<br>OR<br>• Mediators included in model
Analysis	Good/Definitely Low Risk of Bias<br>• Quantitative results presented (e.g., effect estimates with standard errors or confidence limits, not "significant/non-significant")<br>• Missing data noted and addressed in an appropriate fashion<br>• Descriptive information (time-series) about the outcome and exposure presented<br>• Includes sensitivity and/or a priori effect measure modification analyses, including evaluation of multiple lag periods<br>• No deficiencies in analysis evident
	Adequate<br>• Quantitative results presented (e.g., effect estimates with standard errors or confidence limits, not "significant/non-significant")<br>• Missing data noted and addressed in an appropriate fashion<br>• Descriptive information (time-series) about the exposure is presented, but some information may not be provided<br>• Includes sensitivity and/or a priori effect measure modification analyses, but does not include all important sensitivity analyses
	Deficient<br>• Inappropriate control or population selection<br>• Descriptive information about the exposure is not presented<br>• Quantitative results presented without an estimate of random error or only as "significant/non-significant"
	Critically Deficient<br>• Analysis methods are not appropriate for design or data of the study
Selective Reporting	Good/Definitely Low Risk of Bias<br>• All effects are presented either in the main article or supplemental materials

	Adequate<br>• All main effects are presented either in the main article or supplemental material, but sensitivity analyses and effect measure modification results may not be
	Deficient<br>• Not all main effects are presented
	Critically Deficient<br>• Only statistically significant effects are presented<br>OR<br>• No results presented for analyses discussed in methods
Sensitivity	Adequate<br>• Sufficient variability in drought exposure levels to be able to detect an effect should one exist
	Deficient<br>• Insufficient variability in drought exposure levels to be able to detect an effect should one exist
Overall Study Confidence	High<br>• Generally good across domains, and adequate for sensitivity domain
	Medium<br>• Generally adequate across domains
	Low<br>• One or more deficiencies across domains
	Uninformative<br>• Critically deficient in any one of: participant selection, outcome ascertainment, exposure ascertainment, confounding, or analysis<br>OR<br>• Multiple deficiencies across domains and critically deficient in selective reporting domain

Table S3: Narrative Summaries

Study	Narrative Summary
Soverow et al., 2009	This study by Soverow et al. [40] investigated the relationship between meteorological conditions, including ambient temperature, humidity (measured as dew point temperature), and precipitation, and reported human cases of WNV in the United States from 2001 to 2005. Using human WNV incidence data from 17 states with large case numbers (n = 16,298 total cases), the authors conducted a case-crossover study in which each WNV case served as its own control and the effects of meteorological variables were analyzed at the county level They found that higher weekly maximum temperatures and weekly cumulative temperatures were significantly associated with a 35-83% in-crease in WNV incidence within a lag period of one month. Additionally, elevated mean weekly dew point temperatures were associated with a 9-38% rise in cases over the fol-lowing three weeks. Heavy precipitation on at least one day within a week also contributed to a 29-66% increase in WNV incidence during that same week, as well as during the subsequent two weeks. Furthermore, a

	20-mm increase in cumulative weekly precipitation was associated with a 4-8% rise in human WNV cases over the following 1-2 weeks. Overall, the findings indicate that warmer temperatures, higher humidity, and heavy precipitation enhance the relative rate of human WNV infection, regardless of season.
Shaman et al., 2010	This study by Shaman et al. [37], used data from the state of Colorado, divided into east (riparian plains with a hotter, drier climate) and west (alpine mountains with a moderate cooler, wetter climate) due to climatic factors such as temperature, vegetation, and soil humidity. Mosaic model simulations of root zone soil moisture (RZSM) provided the drought-related variable tested. Human case data of both WNF and WNND between the years 2002-2007 were obtained from the Colorado Department of Public Health and Environment's Communicable Disease Epidemiology Program. Generalized linear models using simultaneous autoregression (SAR GLM) were used to analyze the relationship between Mosaic hydrology RZSM and incidence of human cases. It was found that in Colorado, local soil moisture conditions are associated with human incidence of WNV (including both WNF and WNND). In eastern Colorado, wetter than usual springs and drier than usual summers predict an increased number of human WNV cases. In western Colorado, drier than usual spring and summer conditions appear to increase the risk of human WNV infection.
Tran et al., 2014	The study by Tran et al. [43], included the countries Albania, Algeria, Bosnia and Herzegovina, Bulgaria, Croatia, France, Greece, Hungary, Israel, Italy, Kosovo, Macedonia, Montenegro, Morocco, occupied Palestinian territory, Portugal, Romania, Russia, Serbia, Spain, and Tunisia. Data on confirmed human cases of WNND reported in Europe between 2002 and 2013 were gathered from multiple sources, including the MED-LINE database, Embase, Scopus, the Global Infectious Diseases and Epidemiology Online Network, ProMED for central Europe, the Framework Programme for Research and Technological Development, and the European Centre for Disease Prevention and Control. It is not specified how many of these cases report West Nile neurological dis-ease, as opposed to WNF. Univariate analyses were conducted to test associations between explanatory environmental variables and WNND incidence, and significant variables were tested further for co-linearity. A multivariate logistic regression model was used to analyze the association between district-level annual human case incidence probability and modified normalized difference water index (MNDWI; a remote-sensing index that indicates surface moisture and presence of water bodies), along with other district-level ecological and climatic variables, including the population, the presence of wetlands, the presence of birds' migratory routes, temperature anomalies, and the occurrence of a WNND outbreak during the previous year. Within the EU, the nomenclature of territorial units for statistics classification (NUTS) was used to define districts, and outside of the EU, districts were defined using the Global Administrative Unit Layers (GAUL) project. The probability of WNV infection in the EU and its neighboring countries can be explained as a function of average MNDWI within the 21st MODIS 8-day period (June 9-16); when controlling for the covariates, there was a significant positive association between MNDWI and WNND infection probability.
Marcantonio et al., 2015	The study by Marcantonio et al. [7] explored the environmental factors influencing the incidence of WNF in humans across Europe, western Asia, and

	northern Africa. Using data from the ECDC from 2010 to 2012, the authors analyzed associations between WNF incidence and a range of environmental variables, including land surface temperature, precipitation, vegetation and water indices, land use, and human population density (represented by night-time light intensity). The study employed linear mixed-effects models (LMMs) to account for spatial and temporal variations and con-ducted multi-model inference to select the best predictors of WNF incidence. The key findings indicated that high precipitation in late winter/early spring, high summer temperatures, summer drought (measured as NDWI from April to July), irrigated croplands, and highly fragmented forests were significantly associated with WNF out-breaks. Limitations included the challenges of distinguishing true negative cases from underreported areas and the difficulty of capturing all environmental variables comprehensively at such a large spatial scale.
Skaff and Cheruvelil, 2016	The study by Skaff and Cheruvelil [38] examined correlations between WNV transmission, semi-permanent and permanent wetlands, mosquito species and drought conditions across 17 states in the Northeast and upper Midwest of the US. The abundance of permanent and semi-permanent wetlands was calculated at the county level in all states as a percentage of wetland area relative to the total area of the county. Hydrological connectivity, or the connection of wetlands via streams, was also assessed. County-level drought conditions from 2001 through 2012 were determined using the Palmer Hydrological Drought Index and annual WNV cases in humans (combined WNF and WNND) were obtained from the CDC ArboNet database. Mosquito species distribution was broadly defined as *Culex pipiens* and *Culex restuans* in states west of the Mississippi river and *Culex tarsalis* to the east. The authors found that wetland size had more of an effect on *Cx. tarsalis*, while *Cx. pipiens* and *Cx. restuans* were more influenced by wetland connectivity. For *Cx. tarsalis*, the authors also determined that counties with a large proportion of semi-permanent wetlands had significantly more WNV cases during years that were transitioning from non-drought to drought relative to counties with a lower percentage of semi-permanent wetlands, suggesting that reduced wetland habitat may be increasing contact between mosquitoes and birds, leading to increased WNV transmission. However, this trend was not observed in consecutive drought years and the authors suggest this may be due to increased avian WNV immunity following a year of increased transmission. A similar trend of increased WNV transmission in counties with more semi-permanent wetlands entering drought conditions was not observed for counties designated as *Cx. pipiens* areas. This is possibly due to the utilization of more stable urban wetlands by this mosquito. In some cases, drought had a positive effect on human WNV incidence because *Cx. tarsalis* counties with a high proportion of semi-permanent wetland that experienced non-drought to drought conditions had over 150% higher WNV incidence than *Cx. tarsalis* counties with similar proportions of semi-permanent wetland that had undergone any of the other three climate scenarios (i.e., non-drought to non-drought, drought to drought, or drought to non-drought).
Stilianakis et al., 2016	The study by Stilianakis et al. [42] examined the impact of climate on WNF and WNND cases in Northern Greece as reported to the Hellenic Centre for Disease Control and Prevention (2010-2014). The authors examined the impacts of

	temperature (air and soil), moisture (relative humidity, soil water content, and precipitation), and wind speed. They established baseline average climatic parameters from 1979 through 2008 from 1,104 locations using a 5 km by 5 km climatic grid and four daily time windows (0-6-12-18 UTC). Then the authors assessed years 2010 through 2014 for deviations from these climate parameter baselines, based on weekly maximums and weekly means, and tested for any correlations between these deviations and changes in WNF and WNND cases. In addition to a positive correlation with temperature, the investigators found a significant inverse association with relative humidity during the concurrent week, OR = 0.60 (95% CI: 0.53-0.67), as well as significant inverse associations with relative humidity for lag periods of up to three weeks, lag of one week: OR = 0.67 (0.57-0.76), lag of two weeks: OR = 0.72 (0.64-0.82), and lag of three weeks: OR = 0.83 (0.74-0.94), suggesting that drought conditions could increase WNV transmission risk. While no significant associations were observed between mean weekly soil water content and WNV cases, significant inverse associations were observed for maximum weekly soil water content. The odds of WNV cases decreased with maximum weekly soil water content during the concurrent week: OR = 0.87 (0.83-0.92), after a lag of one week: OR = 0.91 (0.87-0.96), after a lag of two weeks: OR = 0.92 (0.87-0.97), and after a lag of three weeks: OR = 0.92 (0.88-0.97). The authors also used a limited data set of mosquito trapping samples where 19 of 87 traps contained WNV positive mosquitoes, and although they found an inverse relationship between WNV infected mosquitoes and wind speed, no correlation was observed with relative humidity, soil water content, or air temperature. In summary, the investigators found that a lack of water appeared to enhance the transmission of WNV leading to an increase in WNF and WNND cases, possibly due to increased bird and mosquito interactions around limited water sources.
Paull et al., 2017	The study by Paull et al. [8] investigated the dynamics of WNND in the United States, emphasizing the influence of intrinsic factors, such as human immunity, and extrinsic factors, particularly climate conditions, on the incidence of the disease, attempting to develop a predictive model for current and future WNND cases. The multivariate log linear model looked at several key predictors: cumulative incidence, mosquito species-specific temperature-driven relative reproductive rates, total precipitation, drought severity over the 4-month period from May through August, and winter conditions. These predictors were selected based on their biological relevance to the transmission of WNV and correlations with WNV risk identified in prior studies. The researchers used county-level weather data to create state-wide averages weighted by the number of recorded WNND cases. The model accounted for variations in immunity by including a term for cumulative incidence. The study finds that both drought and human immunity were important in predicting WNND incidence. Drought conditions were correlated with increased infection prevalence among key mosquito vectors without affecting their abundance, suggesting that drought may alter transmission dynamics through mechanisms such as changes in host-vector interactions. Future projections indicate increases in drought that could nearly double the average burden of WNND by the mid-21st century.

Ukawuba and Shaman, 2018	The study by Ukawuba and Shaman [41] examined the influence of meteorological and hydrological conditions on annual WNV cases in west Texas. The methodology relied on a Bayesian model averaging framework to compare monthly environmental conditions, such as temperature, precipitation, specific humidity, and soil moisture to predict WNV infection in humans. Leave-one-year-out temporal cross validation was conducted to ensure that the results were not representative of only a single year's data out of the 15-year study period. The investigators found that wetter and more humid spring conditions, followed by drier and cooler summers, are strongly associated with increased WNV infections. Soil moisture, particularly in March, June, and August, emerged as an important predictor, out-performing precipitation in predicting WNV case variability. The findings suggest that wet spring conditions facilitate mosquito reproduction and enhance interactions with avian hosts, which is crucial for WNV circulation. The study highlights that cooler summer temperatures may inadvertently increase human exposure to mosquitoes due to outdoor activities during optimal mosquito feeding times.
Smith et al., 2020	The study by Smith et al. [39] aimed to predict future risk of WNV infection in humans in Nebraska by analyzing monthly precipitation, temperature, standardized precipitation index, and standardized precipitation and evapotranspiration index data. Using generalized additive models with a negative binomial distribution, researchers explored various combinations of temperature and drought data, lagged by up to 36 months to determine which factors influenced WNV infection. Annual counts of human cases of WNV per county were used and the effect of previous WNV exposure was incorporated by using cumulative incidence rates, which were determined using total previous cases per county, per year, per 100,000 population based on CDC's Arbonet data from 2002 to 2018. County-level cumulative incidence of human WNV cases per 100,000 people in Nebraska ranged from 0 to greater than 1,000 for 2018. Data from 2002 to 2011 were used to create and train models, with data from 2012-2018 and subsequent years serving as out-of-sample predictions. Models indicated that the strongest predictors of WNV cases were wet years followed by a dry year with warm temperatures. The models out-performed random chance and naïve persistence models in predicting which counties would experience cases. Notably, the study's model predictions suggested that without drought conditions, WNV cases could have been reduced by 26%; without warm temperatures, they could have been reduced by 29%; and with neither factor present, cases could have been decreased by 45%.
